# Addition of polygenic risk score to a risk calculator for prediction of breast cancer in US Black women

**DOI:** 10.1186/s13058-023-01748-8

**Published:** 2024-01-02

**Authors:** Gary R. Zirpoli, Ruth M. Pfeiffer, Kimberly A. Bertrand, Dezheng Huo, Kathryn L. Lunetta, Julie R. Palmer

**Affiliations:** 1 Slone Epidemiology Center at Boston University, Boston, MA USA; 2 Biostatistics Branch, Division of Cancer Epidemiology and Genetics, National Cancer Institute, Bethesda, MD USA; 3 Department of Medicine, Boston University Chobanian & Avedisian School of Medicine, Boston, MA USA; 4 Department of Public Health Sciences, The University of Chicago, Chicago, IL USA; 5 Center for Clinical Cancer Genetics & Global Health, The University of Chicago, Chicago, IL USA; 6 Department of Biostatistics, Boston University School of Public Health, Boston, MA USA; 7 Division of Cancer Epidemiology and Biostatistics, National Cancer Institute, Bethesda, USA

**Keywords:** Risk prediction, Breast cancer, African American, Polygenic risk score

## Abstract

**Background:**

Previous work in European ancestry populations has shown that adding a polygenic risk score (PRS) to breast cancer risk prediction models based on epidemiologic factors results in better discriminatory performance as measured by the AUC (area under the curve). Following publication of the first PRS to perform well in women of African ancestry (AA-PRS), we conducted an external validation of the AA-PRS and then evaluated the addition of the AA-PRS to a risk calculator for incident breast cancer in Black women based on epidemiologic factors (BWHS model).

**Methods:**

Data from the Black Women’s Health Study, an ongoing prospective cohort study of 59,000 US Black women followed by biennial questionnaire since 1995, were used to calculate AUCs and 95% confidence intervals (CIs) for discriminatory accuracy of the BWHS model, the AA-PRS alone, and a new model that combined them. Analyses were based on data from 922 women with invasive breast cancer and 1844 age-matched controls.

**Results:**

AUCs were 0.577 (95% CI 0.556–0.598) for the BWHS model and 0.584 (95% CI 0.563–0.605) for the AA-PRS. For a model that combined estimates from the questionnaire-based BWHS model with the PRS, the AUC increased to 0.623 (95% CI 0.603–0.644).

**Conclusions:**

This combined model represents a step forward for personalized breast cancer preventive care for US Black women, as its performance metrics are similar to those from models in other populations. Use of this new model may mitigate exacerbation of breast cancer disparities if and when it becomes feasible to include a PRS in routine health care decision-making.

**Supplementary Information:**

The online version contains supplementary material available at 10.1186/s13058-023-01748-8.

## Introduction

Breast cancer mortality rates are 40% higher in Black women than White women in the USA, even though incidence rates are approximately the same [1]. Multiple approaches are needed to eliminate this disparity. One such approach is to improve risk prediction tools so that Black women who are at high risk of breast cancer, together with their physicians, are better able to make informed decisions about when to begin mammographic screening, frequency of screening, and use of other screening modalities such as breast MRI. In addition, improved risk prediction for specific subtypes of breast cancer will permit targeted enrollment of Black women into prevention trials for medications for estrogen receptor (ER)-positive or ER-negative breast cancer to increase the likelihood that medications developed will also benefit Black women.

In previous work, we developed and validated an absolute risk prediction model for breast cancer incidence in US Black women [2], among whom there is a higher proportion of ER-negative vs. ER-positive tumors than in US women from other racial groups. This model (Black Women’s Health Study (BWHS) model) used women’s personal and clinical characteristics to predict risk. Its discriminatory accuracy, as measured by the area under the receiver operator characteristics curve (AUC), was modest, with an AUC of 0.58 (95% confidence interval (CI) 0.56–0.59). Although this AUC is on par with measures of discriminatory accuracy for similar risk factor-based models in predominantly White populations [3–6], there is a need for more accurate prediction. Multiple genetic risk variants, individually or combined into a polygenic risk score (PRS), have been shown to significantly improve the discriminatory ability of established risk models based on data from White women [7–13]. Until 2022, attempts to derive and/or validate PRS for breast cancer in women of African ancestry had failed [14–17]. Regardless of whether the PRS had been derived in data from largely European ancestry populations or from smaller studies of African ancestry populations, the per standard deviation odds ratios and AUCs were markedly lower in populations of African ancestry than European ancestry [18], Asian ancestry [19], or Hispanic American [20] populations. Poor performance of PRS-based models likely reflects the greater genetic variation and smaller linkage disequilibrium blocks in individuals of predominantly African ancestry and a smaller number of breast cancer cases with available genome-wide association study (GWAS) data. In 2022, Guo et al. used data from close to 10,000 breast cancer cases and 10,000 controls of African ancestry to derive a PRS (AA-PRS) and conduct internal validation of its predictive performance [21]. The odds ratio per standard deviation was 1.34 (1.27–1.42) and the AUC corresponding to that PRS was 0.58, much closer to the metrics obtained in studies of other populations [18–20].

In the current work, we conducted the first external validation of this AA-PRS. We then evaluated whether and to what extent adding this novel AA-PRS to the BWHS risk factor model would improve prediction of five-year absolute risk of breast cancer in US Black women.

## Methods

### Study population

The BWHS is a prospective cohort study of 59,000 self-identified Black women, aged 21–69 at baseline in 1995, from across the US who enrolled in the study by completing a lengthy baseline questionnaire [22]. Since then, biennial questionnaires that ask about medical history, medication use, and social, reproductive, and lifestyle factors have been used to update information on exposure variables and health events, including incident breast cancer diagnoses. Approximately 30,000 BWHS participants have provided a biospecimen (saliva or blood) that could be used as a source of germline DNA. The eligible study population for this project comprised approximately 6000 BWHS participants for whom genome-wide single-nucleotide polymorphism (SNP) data were available. The study protocol was approved by the Boston University Institutional Review Board.

### Cases

Incident cases of breast cancer in the BWHS were ascertained through questionnaire self-report, linkage with state cancer registries, and death records. Cases were confirmed and tumor characteristics were determined from review of pathology reports and/or state cancer registry data, which have been obtained for over 90% of breast cancer cases. Eligible cases for the present analyses were women who were diagnosed with invasive breast cancer from 1995 through 2019, aged 30–74 at diagnosis, had GWAS data available, and had not been included in derivation of the AA-PRS. In total, there were 922 cases, including 555 with ER-positive and 296 with ER-negative breast cancer; ER status was unknown for the remainder of the cases.

### Controls

Risk set sampling was used to select two controls per case. Controls were free of breast cancer at the time the index case was diagnosed and were matched to cases on year of age and timing of the most recent follow-up questionnaire completed. As with the cases, potential controls had GWAS data available and had not been included in derivation of the AA-PRS. There were 1,844 controls included.

### Risk predictors

The BWHS breast cancer risk prediction model was developed using data from Black women in three large breast cancer case–control studies and then validated in prospective data from the BWHS, as described previously [2]. Model predictors include first-degree family history of breast cancer and prostate cancer, body mass index (BMI) (current and at age 18), menopausal status, bilateral oophorectomy, breast biopsy, oral contraceptive use, age at menarche, ever parous, and breastfeeding. The BWHS model also includes age interactions with family history of breast cancer, breast biopsy, and age at menarche, and an interaction of menopausal status with current BMI.

Development of the AA-PRS by Gao et al. has been described elsewhere [21]. Separate PRS were developed for each of ER-positive and ER-negative breast cancer as the weighted linear combination of a PRS developed in data from women of African ancestry and a PRS previously developed in data from women of European ancestry. A PRS for overall breast cancer was then constructed by averaging the ER-positive and ER-negative PRS, weighted by the study subtype proportions. BWHS samples from breast cancer cases and controls had been previously genotyped on the Illumina MEGA array and were imputed to the same reference panel as the samples in Gao et al. Genotype or imputation values for the variants identified by Gao et al. were used for calculation of a PRS in each of 922 BWHS cases and 1844 controls after removal of 23 SNPs with low imputation scores in BWHS data. There were 56,920 variants included in the PRS for breast cancer overall, 29,299 for the ER-positive breast cancer PRS, and 28,392 for the ER-negative breast cancer PRS. None of the 922 BWHS cases and 1844 controls contributed to the work by Gao et al., thus ensuring an external validation sample.

Principal components of the BWHS genotype data were calculated with smartpca in the EIGENSOFT package [23], after pruning SNPs in high linkage disequilibrium (pairwise correlation > 0.1) and removing SNPs with minor allele frequency < 0.02 and more than 0.5% missing. We assessed the associations of the first 10 principal components with breast cancer risk by including them jointly in a logistic regression model and retained those associated with *p* < 0.05. Principal components from the study population rather than principal components from the data analyses by Gao et al. were used because there may have been allele frequency differences in the two populations stemming from their different geographic distributions.

### Statistical methods

#### External validation of AA-PRS

Associations between PRS and invasive breast cancer risk in the BWHS, overall and by ER status, were evaluated in conditional logistic regression analyses, with and without adjustment for principal components associated with breast cancer risk (1, 3, and 7). Percentile categories were constructed based on the distribution of PRS in the controls (≤ 10%, 10-20%, 20–40%, 40–60%, 60–80%, 80–90%, and > 90%). Odds ratios (ORs) and corresponding 95% CIs were computed for percentiles of the PRS with 40–60% as the reference category. Additionally, ORs and 95% CIs for a one standard deviation (SD) increase in continuous PRS were calculated. For ER-specific analyses, we used the ER-specific PRS from Gao et al. rather than the overall PRS. All statistical analyses were performed using SAS 9.4 (Cary, NC).

#### Addition of PRS to the risk factor-based BWHS prediction model

We first applied the BWHS risk prediction calculator to derive a five-year absolute risk estimate for each participant. We log-transformed the absolute risks and then estimated the concordance index (c-index, which for ease of exposition we also refer to as “AUC”), accommodating the matched study design, for the risk of invasive breast cancer based on the log absolute risks derived from the BWHS model alone [24]. We then similarly calculated the AUC for the PRS. Missing data was addressed with multiple imputation (IVEware 0.3). C-indices and bootstrapped standard errors were calculated for each of 10 imputed datasets and combined with Rubin’s rules [25, 26]. We next examined the correlation of the PRS with the BWHS risk estimates. We then computed a score for the BWHS model plus PRS, using a leave-one-out approach. We left out one matched set at a time and fit a conditional logistic regression to the remaining sets, including terms for the BWHS model and PRS. The parameter estimates from this regression were then applied to the coefficients of the participants in the set left out. This procedure was continued for the remaining sets and an AUC was calculated for this score. Comparable AUCs were also calculated separately for ER-positive and ER-negative breast cancer and in women under age 45 and older women.

We calculated net reclassification improvement (NRI) following the approach by Pencina et al. for case–control studies, as follows [27]. We fit two logistic regression models to the case–control data: one including the log-transformed BWHS risk estimates and one including both the log-transformed BWHS risk estimates and the PRS. We then adjusted the intercepts of the models by adding $\mathrm{log}\{2\rho /(1-\rho )\}$, where 2 corresponds to the control to case ratio in the study and the constants $\rho$ were the 5-year age-specific breast cancer incidence rates for 2000–2016 for non-Hispanic Black women from the NCI SEER program. This intercept adjustment ensures that the logistic regression models based on BWHS and BWHS plus PRS are calibrated to predict 5-year age-specific breast cancer incidence in US Black women. We then used risk cut points 1.66% and 2.5% to calculate the NRI [28]. We calculated the NRI for each imputed dataset and averaged their values to obtain the final NRI estimate. The variance was computed using Rubin’s rules [25]. For presenting the reclassification table (Table 3), we averaged the probability estimates for each woman over all imputations.

## Results

### External validation of AA-PRS

**Fig. 1:**
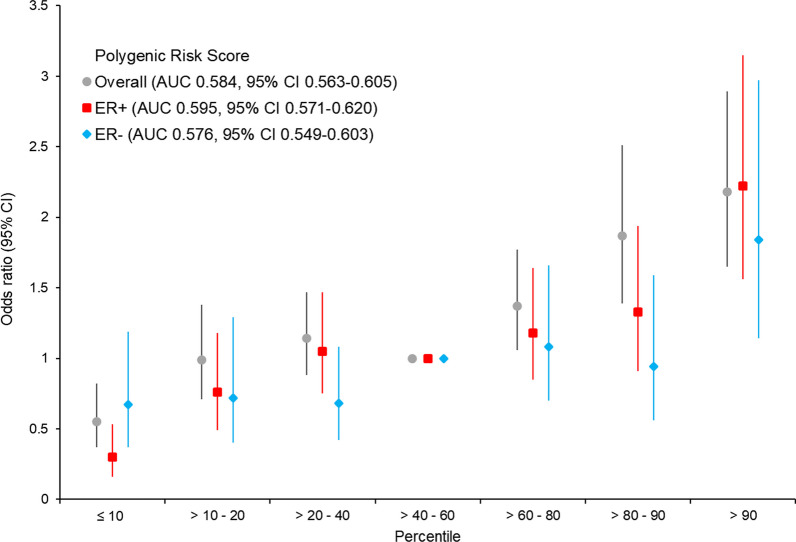
Odds ratios for associations of polygenic risk score (PRS) quantiles with risk of invasive breast cancer, overall, and by estrogen receptor status (ER+ , ER−). Reference category is middle quintile

As shown in Fig. 1 and Additional file 1: Table S1, there were 922 BWHS breast cancer cases and 1,844 controls available for validation of the PRS. Because association estimates were almost identical in analyses with and without adjustment for principal components, we present results without such adjustment. For overall breast cancer, the OR per SD was 1.42 (95% CI 1.31–1.54) and the AUC was 0.584 (95% CI 0.563–0.605). The OR for women in the top decile of the PRS relative to women at average risk (40–60th percentile) was 2.18 (95% CI 1.65–2.89). The per standard deviation OR for ER-positive breast cancer, based on 555 cases and using the ER-positive specific PRS, was 1.51 (95% CI 1.36–1.68), with an AUC of 0.595 (95% CI 0.571–0.620). For ER-negative breast cancer, the comparable OR was 1.35 (95% CI 1.18–1.54) and the AUC was 0.576 (95% CI 0.549–0.603).

### Addition of PRS to the BWHS risk factor-based prediction model

Table 1 shows the factors that were included in the BWHS absolute risk prediction calculation, with prevalence of each factor by case–control status. Compared with controls, cases were more likely to have a first-degree family history of breast cancer and to have had a breast biopsy, and were less likely to have been overweight or obese at age 18 or to have had a bilateral oophorectomy. Cases had a higher mean overall PRS than controls (0.23 vs −0.12). They also were estimated to have a higher five-year absolute risk (1.46% in cases vs 1.32% in controls). There was little correlation between the BWHS predicted risks and the PRS, with a Pearson correlation coefficient of 0.039.

**Table 1:** Participant characteristics, including factors in the BWHS risk prediction model, by case–control status

	Cases (*n* = 922) (%)	Controls (*n* = 1844) (%)
First-degree family history of breast cancer		
Age < 50 years		
No	275 (79.3)	575 (82.9)
Relative diagnosed age ≥ 50 years	46 (13.3)	100 (14.4)
Relative diagnosed age < 50 years or two relatives	26 (7.5)	19 (2.7)
Age ≥ 50 years		
No	416 (72.3)	924 (80.3)
Relative diagnosed age ≥ 50 years	109 (19.0)	185 (16.1)
Relative diagnosed age < 50 years or two relatives	50 (8.7)	41 (3.6)
First-degree family history of prostate cancer		
No	728 (79.0)	1509 (81.8)
Yes	194 (21.0)	335 (18.2)
Age < 50 years		
Age at menarche < 14 years	295 (85.3)	551 (80.1)
Age at menarche ≥ 14 years	51 (14.7)	137 (19.9)
Age ≥ 50 years		
Age at menarche < 14 years	483 (84.0)	887 (77.6)
Age at menarche ≥ 14 years	92 (16.0)	256 (22.4)
Breastfeeding (parous women only)		
Never	382 (53.1)	840 (57.3)
Ever	337 (46.9)	625 (42.7)
Oral contraceptive use		
Never or < 5 years	547 (59.3)	1266 (68.7)
≥ 5 years	375 (40.7)	578 (31.3)
Bilateral oophorectomy		
No	818 (88.7)	1625 (88.1)
Yes	104 (11.3)	219 (11.9)
BMI at age 18 years, kg/m^2^		
< 25	830 (91.3)	1562 (86.3)
≥ 25	79 (8.7)	247 (13.7)
Premenopausal		
BMI < 30	206 (64.2)	423 (62.9)
BMI ≥ 30	115 (35.8)	249 (37.1)
Postmenopausal		
BMI < 30	214 (57.1)	487 (58.7)
BMI ≥ 30	161 (42.9)	342 (41.3)
Age < 50 years		
Never biopsy or benign breast disease	247 (71.2)	534 (76.9)
Ever biopsy or benign breast disease	100 (28.8)	160 (23.1)
Age ≥ 50 years		
Never biopsy or benign breast disease	300 (52.2)	702 (61.0)
Ever biopsy or benign breast disease	275 (47.8)	448 (39.0)
Mean age (SD)	52.7 (9.3)	52.7 (9.3)
Polygenic risk score, mean (SD)	0.23 (1.00)	− 0.12 (0.98)
BWHS 5-year breast cancer risk percent, mean (SD)	1.46 (0.67)	1.32 (0.57)

As shown in Table 2, breast cancer risk prediction was improved with the addition of the AA-PRS. The AUC for overall breast cancer was 0.577 from the BWHS risk prediction model alone; the addition of the AA-PRS increased it to 0.623, an increase of 0.046 units. Increases in the AUCs with the addition of a PRS were 0.033 for ER-positive breast cancer and 0.062 for ER-negative breast cancer, and were 0.062 and 0.044, respectively, among women age <45 and age ≥45.

**Table 2:** Discriminatory accuracy of BWHS risk model alone, polygenic risk score (PRS) alone, and model that combines both

	AUC (95% CI)	Increase in AUC from combined versus BWHS model (95% CI)	*p*-value
All invasive breast cancers (*N* = 922)			
BWHS model only	0.577 (0.556–0.598)		
PRS only	0.584 (0.563–0.605)		
BWHS model + PRS	0.623 (0.603–0.644)	0.046 (0.023–0.069)	< 0.0001
ER-positive breast cancer (*N* = 555)			
BWHS model only	0.594 (0.572–0.617)		
PRS only	0.595 (0.571–0.620)		
BHS model + PRS	0.627 (0.603–0.651)	0.033 (0.008–0.057)	0.0099
ER-negative breast cancer (*N* = 296)			
BWHS model only	0.536 (0.509–0.563)		
PRS only	0.576 (0.549–0.603)		
BWHS model + PRS	0.597 (0.568–0.627)	0.061 (0.031–0.093)	0.0001
Age < 45 years at breast cancer diagnosis or control selection (*N* = 208)			
BWHS model only	0.546 (0.507–0.586)		
PRS only	0.587 (0.551–0.623)		
BWHS model + PRS	0.608 (0.569–0.647)	0.062 (0.022–0.101)	0.0025
Age ≥ 45 years at breast cancer diagnosis or control selection (*N* = 714)			
BWHS model only	0.586 (0.563–0.609)		
PRS only	0.583 (0.559–0.608)		
BWHS model + PRS	0.630 (0.606–0.653)	0.044 (0.020–0.068)	0.0003

Table 3 shows classification of predicted risk by the two models for cases and controls. The net reclassification index was 9.2%, based on the sum of a classification improvement of 11.8% in cases and −2.6% in controls.

**Table 3:** Reclassification for BWHS predicted 5-year risk versus BWHS + PRS predicted 5-year risk

Cases	BWHS + PRS			Total
	< 1.66%	1.66–< 2.5%	≥ 2.5%	
BWHS model only				
< 1.66%	503	94	23	620
1.66–< 2.5%	72	134	74	280
≥ 2.5%	2	8	12	22
Total	577	236	109	922

Controls	BWHS + PRS			Total
	< 1.66%	1.66–< 2.5%	≥ 2.5%	
BWHS model only				
< 1.66%	1146	126	13	1285
1.66–< 2.5%	185	245	106	536
≥ 2.5%	4	9	10	23
Total	1335	380	129	1844

Net reclassification improvement (95% CI) = 0.092 (0.065–0.119)

For cases, reclassification = 0.118; for controls, reclassification = − 0.026

## Discussion

Polygenic risk scores developed in women of European ancestry have not performed as well in women of African ancestry [14, 29, 30]. Gao et al. moved the field forward by using GWAS data from multiple studies of African ancestry women to develop and test a PRS for breast cancer overall and for ER-specific breast cancer, producing for the first time a PRS with discriminatory accuracy close to what has been observed in other populations [18, 20]. Here, we present results of the first external validation of that AA-PRS, with an AUC of 0.58 and OR of 1.41 for each standard deviation unit of risk, similar to the AUC 0.58 and OR 1.34 from the previously published internal validation [21]. Until now, the use of a PRS for improved breast cancer risk prediction would have increased racial disparities in breast cancer because women of African ancestry would receive little benefit, if any, from a PRS derived from predominantly European ancestry populations. Now, with external validation of this AA-PRS in a large cohort of US Black women, there is finally a validated PRS that can be used in this population.

The best performing PRS for women of European ancestry was developed in the Breast Cancer Association Consortium (BCAC). This 313-SNP PRS had an AUC of 0.630 (95% CI 0.628–0.651) and an OR per SD unit of PRS of 1.61 (95% CI 1.57–1.65) for overall breast cancer [18]. In a collaborative study of US Latina women and Latin American women, Shieh et al. constructed a 180-SNP PRS and reported an AUC of 0.63 (95% CI 0.62–0.64) in internal validation [20]. The OR per SD unit increase was 1.58 (95% CI 1.52–1.64) and the OR for those above the 90th percentile of PRS compared to women in the 40–60th percentile group was 2.10 (95% CI 1.85–2.39). In the present study of women of African ancestry, ORs for above the 90th percentile versus the 40–60th percentile group were 2.18 for overall breast cancer, 2.22 for ER+ breast cancer, and 1.84 for ER- breast cancer.

We also examined the utility of adding this AA-PRS to the BWHS breast cancer risk prediction model, which was previously developed and validated in data from US Black women [2]. For all invasive breast cancer, the addition of the AA-PRS improved discriminatory accuracy, increasing the AUC from 0.58 (BWHS model alone) to 0.62. It was not possible to estimate calibration of the combined model because of the case–control design and lack of prospective cohort data with genetic information for validation, but in the original validation of the BWHS breast cancer risk prediction model, the ratio of expected numbers of cancers calculated from the model and observed numbers of cancers was 1.01 (0.95–1.07), indicating excellent overall calibration [2]. We postulate that a new, combined absolute breast cancer risk model will likely also be well calibrated, but that would need to be demonstrated in data with prospective follow-up. The addition of a validated 313-SNP PRS to various breast cancer risk prediction tools has been evaluated in multiple populations of European ancestry women [11–13, 18, 31–35]. In an Australian prospective cohort study, the addition of the 313-SNP PRS improved the AUC from 0.57 to 0.62 for the IBIS model and from 0.56 to 0.62 for the BOADICEA model [11]. In a combined analysis of 15 cohorts of European ancestry women, the addition of the 313-SNP PRS to the iCARE-Lit model improved the AUC from 0.56 to 0.64 in women under 50 years of age and from 0.57 to 0.64 in women 50 years and older [12]. In data from the Nurses’ Health Study and Nurses’ Health Study II, the addition of a PRS improved the AUC for the BCRAT model from 0.56 to 0.61 in premenopausal women, from 0.55 to 0.61 in postmenopausal women not using hormone therapy, and from 0.58 to 0.62 in postmenopausal women using hormone therapy [36]. Results concerning magnitude of the AUC and increase in AUC after the addition of the PRS in the current study of African ancestry women are very similar to results from these large studies of European ancestry women.

Prior evaluation of the addition of a PRS to breast cancer risk prediction models in women of African ancestry has been limited. Allman et al. [39] used data from the Women’s Health Initiative to calculate AUCs after the addition of a PRS to two established risk prediction models that included epidemiologic factors only, the BCRAT [37] and the IBIS model [38]. The 75-SNP PRS included SNPs associated with breast cancer risk in data from women of European ancestry. The addition of the PRS increased the AUC in both models, from 0.56 to 0.59 in the BCRAT and from 0.51 to 0.55 in the IBIS model. Most recently, Tshiaba et al. have evaluated the addition of a cross-ancestry PRS [40] to the IBIS model in data from the Women’s Health Initiative and the UK Biobank [41]. Across all ancestry groups, the addition of the PRS to the IBIS model increased the AUC for prediction of risk in the next five years from 0.56 to 0.65 in the WHI and from 0.57 to 0.63 in the UK Biobank. However, performance was markedly worse in women of African ancestry; the AUC increased from 0.55 to 0.57 in WHI data. There were too few breast cancer cases (*n* = 19) for five-year risk prediction among Black/Black British women in the UK Biobank.

The present study included 296 ER-negative and 555 ER-positive invasive breast cancer cases, allowing for validation of the previously published ER-specific PRS and examination of whether adding an ER-specific PRS improves discriminatory accuracy. The BWHS risk calculator alone had a higher discriminatory accuracy for ER-positive versus ER-negative breast cancer in the validation data set (AUC 0.59 and 0.54, respectively), similar to what has been found in other studies that evaluated epidemiologic risk models separately for ER-positive and ER-negative breast cancer [2, 42–44]. This is not surprising because many of the factors included in the models (e.g., hormone replacement therapy, age at menarche, high body mass index after menopause, bilateral oophorectomy) are more strongly associated with ER+ breast cancer, which has a hormonal etiology. Improvements in AUC with the addition of an ER-specific PRS were somewhat greater for ER-negative breast cancer, with an increase in 0.061 units versus an increase in 0.033 units for ER-positive cancer. This finding demonstrates the value of identifying common genetic variants associated with risk of ER-negative breast cancer in women of African ancestry.

A limitation of our study is the lack of data on mammographic density and endogenous hormone levels, both of which are related to breast cancer risk. When available, these factors could improve discriminatory accuracy for the purposes of shared decision-making on use of anti-estrogenic medications for women at high risk of ER-positive breast cancer and for eligibility to be included in future breast cancer prevention trials [36, 45, 46]. Data on hormone levels will not be useful for purposes of shared decision-making on timing and type of breast cancer screening in the foreseeable future due to the high costs of the assays. Incorporation of mammographic density data or data on texture features of the breast beyond density will be useful for women who have already started screening, but not for those, including many young women, who have not yet had their first mammogram.

## Conclusions

In summary, by combining estimates from the previously validated BWHS breast cancer risk prediction model with the newly validated AA-PRS, we now have a combined model that provides discriminatory accuracy higher than the BWHS model alone and similar in magnitude to combined models in women of European ancestry. Cross-ancestry models are being put forth as valuable for multiple ancestral populations, but, to date, show relatively poor performance in African ancestry populations [41]. To develop a cross-ancestry PRS that works well for all major population groups, it will be necessary to have larger numbers of cases and controls from African ancestry populations and from other populations currently underrepresented in genetics research. Until then, the combined model developed here represents a critical step forward for personalized breast cancer preventive care for US Black women and has the potential to mitigate exacerbation of racial disparities in breast cancer as PRS become more widely used in clinical settings.

### Supplementary Information

**Additional file 1. Table S1**. Association of previously derived polygenic risk score (PRS) with risk of invasive breast cancer in US Black women.

## Data Availability

The datasets used and/or analyzed during the current study are available from the corresponding author on reasonable request.

## Abbreviations

AA-PRS: African ancestry polygenic risk score
AUC: Area under the receiver operator characteristics curve
BMI: Body mass index
BWHS: Black Women's Health Study
CI: Confidence interval
ER: Estrogen receptor
GWAS: Genome-wide association study
IRB: Institutional review board
OR: Odds ratio
PRS: Polygenic risk score
SD: Standard deviation
SNP: Single-nucleotide polymorphism
