# Supplementary material for: Addition of polygenic risk score to a risk calculator for prediction of breast cancer in US Black women
Source: Breast Cancer Res. 2024 Jan 2;26:2. doi: 10.1186/s13058-023-01748-8 (PMC10763003; doi:10.1186/s13058-023-01748-8)
Supplement: Supplementary file 1 — Additional file 1. Table S1. Association of previously derived polygenic risk score (PRS) with risk of invasive breast cancer in US Black women. [file 13058_2023_1748_MOESM1_ESM.docx]

| **Table S1. Association of previously derived polygenic risk score (PRS) with risk of invasive breast cancer in U.S. Black women** | | | | | | | | | |
| --- | --- | --- | --- | --- | --- | --- | --- | --- | --- |
|  | Overall breast cancer | | | ER-positive breast cancer^1^ | | | ER-negative breast cancer^2^ | | |
|  | Cases | Controls | Odds ratio | Cases | Controls | Odds ratio | Cases | Controls | Odds ratio |
| Percentile^3^ | N (%) | N (%) | (95% CI) | N (%) | N (%) | (95% CI) | N (%) | N (%) | (95% CI) |
| ≤ 10 | 42 (4.6) | 185 (10.0) | 0.55 (0.37-0.82) | 16 (2.9) | 111 (10.0) | 0.30 (0.16-0.53) | 21 (7.1) | 62 (10.5) | 0.67 (0.37-1.19) |
| 10 - ≤ 20 | 71 (7.7) | 180 (9.8) | 0.99 (0.71-1.38) | 37 (6.7) | 111 (10.0) | 0.76 (0.49-1.18) | 21 (7.1) | 55 (9.3) | 0.72 (0.40-1.29) |
| 20 - ≤ 40 | 165 (17.9) | 371 (20.1) | 1.14 (0.88-1.47) | 103 (18.6) | 221 (19.9) | 1.05 (0.75-1.47) | 39 (13.2) | 116 (19.6) | 0.68 (0.42-1.10) |
| 40 - ≤ 60 | 151 (16.4) | 379 (20.6) | 1.00 (ref) | 101 (18.2) | 223 (20.1) | 1.00 (ref) | 64 (21.6) | 123 (20.8) | 1.00 (ref) |
| 60 - ≤ 80 | 196 (21.3) | 365 (19.8) | 1.37 (1.06-1.77) | 115 (20.7) | 221 (19.9) | 1.18 (0.85-1.64) | 64 (21.6) | 114 (19.3) | 1.08 (0.70-1.66) |
| 80 - ≤ 90 | 134 (14.5) | 179 (9.7) | 1.87 (1.39-2.51) | 68 (12.3) | 112 (10.1) | 1.33 (0.91-1.94) | 30 (10.1) | 63 (10.6) | 0.94 (0.56-1.59) |
| > 90 | 163 (17.7) | 185 (10.0) | 2.18 (1.65-2.89) | 115 (20.7) | 111 (10.0) | 2.22 (1.56-3.15) | 57 (19.3) | 59 (10.0) | 1.84 (1.18-2.97) |
|  |  |  |  |  |  |  |  |  |  |
| per 1 SD of PRS | 922 | 1844 | 1.42 (1.31-1.54) | 555 | 1110 | 1.51 (1.36-1.68) | 296 | 592 | 1.35 (1.18-1.54) |
|  |  |  |  |  |  |  |  |  |  |
| AUC |  |  | 0.584 (0.563-0.605) |  |  | 0.595 (0.571-0.620) |  |  | 0.576 (0.549-0.603) |
| Odds ratios from conditional logistic regression analyses. CI denotes confidence interval.  ^1^PRS derived for ER-positive breast cancer.  ^2^PRS derived for ER-negative breast cancer.  ^3^Percentile determined from PRS distribution among each control group. | | | | | | | | | |
